# The implicit power motive predicts action selection

**DOI:** 10.1007/s00426-016-0768-z

**Published:** 2016-03-23

**Authors:** Peter F. Stoeckart, Madelijn Strick, Erik Bijleveld, Henk Aarts

**Affiliations:** 1 Department of Psychology, Utrecht University, P.O. Box 126, 3584 CS Utrecht, The Netherlands; 2 Behavioural Science Institute, Radboud University, Nijmegen, The Netherlands

## Abstract

Previous research has indicated that implicit motives can reliably predict which behaviors people select or decide to perform. However, so far, the question of how these motives are able to predict this action selection process has received little attention. Based on ideomotor theory, we argue that implicit motives can predict action selection when an action has become associated with a motive-congruent (dis)incentive through repeated experiences with the action-outcome relationship. This idea was investigated by examining whether the implicit need for power (*n*Power) would come to predict action selection (i.e., choosing to press either of two buttons) when these actions had repeatedly resulted in motive-congruent (dis)incentives (i.e., submissive or dominant faces). Both Studies 1 and 2 indicated that participants became more likely to select the action predictive of the motive-congruent outcome as their history with the action-outcome relationship increased. Study 2 indicated that this effect stemmed from both an approach towards incentives and an avoidance of disincentives. These results indicate that implicit motives (particularly the power motive) can predict action selection as a result of learning which actions yield motive-congruent (dis)incentives. Our findings therefore offer a model of how implicit motives can come to predict which behaviors people select to perform.

**Electronic supplementary material:**

The online version of this article (doi:10.1007/s00426-016-0768-z) contains supplementary material, which is available to authorized users.

## Introduction

A major part of everyday human behavior consists of making decisions. When making these decisions, people often rely on what motivates them most. Accordingly, human behavior generally originates from an action selection process that takes into account whether the effects resulting from actions match with people’s motives (Bindra, 1974; Deci & Ryan, 2000; Locke & Latham, 2002; McClelland, 1985). Although people can explicitly report on what motivates them, these explicit reports tell only half the story, as there also exist implicit motives of which people are themselves unaware (McClelland, Koestner, & Weinberger, 1989). These implicit motives have been defined as people’s non-conscious motivational dispositions that orient, select and energize spontaneous behavior (McClelland, 1987). Generally, three different motives are distinguished: the need for affiliation, achievement or power. These motives have been found to predict many different types of behavior, such as social interaction frequency (Wegner, Bohnacker, Mempel, Teubel, & Schüler, 2014), task performance (Brunstein & Maier, 2005), and emotion detection (Donhauser, Rösch, & Schultheiss, 2015). Despite the fact that many studies have indicated that implicit motives can direct and control people in performing a variety of behaviors, little is known about the mechanisms through which implicit motives come to predict the behaviors people choose to perform. The aim of the current article is to provide a first attempt at elucidating this relationship between implicit motives (particularly the power motive) and the selection of specific behaviors.

An important tenet underlying most decision-making models and expectancy value approaches to action selection and behavior is that people are generally motivated to increase positive and limit negative experiences (Kahneman, Wakker, & Sarin, 1997; Oishi & Diener, 2003; Schwartz, Ward, Monterosso, Lyubomirsky, White, & Lehman, 2002; Thaler, 1980; Thorndike, 1898; Veenhoven, 2004). Hence, when someone has to select an action from several potential candidates, this person is likely to weigh each action’s respective outcomes based on their to be experienced utility. This ultimately results in the action being selected which is perceived to be most likely to yield the most positive (or least negative) result. For this process to function properly, people would need to be able to predict the consequences of their potential actions.

This process of action-outcome prediction in the context of action selection is central to the theoretical approach of ideomotor learning. According to ideomotor theory (Greenwald, 1970; Shin, Proctor, & Capaldi, 2010), actions are stored in memory in conjunction with their respective outcomes. That is, if a person has learned through repeated experiences that a specific action (e.g., pressing a button) produces a specific outcome (e.g., a loud noise) then the predictive relation between this action and respective outcome will be stored in memory as a common code (Hommel, Müsseler, Aschersleben, & Prinz, 2001). This common code thereby represents the integration of the properties of both the action and the respective outcome into a singular stored representation. Because of this common code, activating the representation of the action automatically activates the representation of this action’s learned outcome. Similarly, the activation of the representation of the outcome automatically activates the representation of the action that has been learned to precede it (Elsner & Hommel, 2001). This automatic bidirectional activation of action and outcome representations makes it possible for people to predict their potential actions’ outcomes after learning the action-outcome relationship, as the action representation inherent to the action selection process will prime a consideration of the previously learned action outcome.

When people have established a history with the action-outcome relationship, thereby learning that a specific action predicts a specific outcome, action selection can be biased in accordance with the divergence in desirability of the potential actions’ predicted outcomes. From the perspective of evaluative conditioning (De Houwer, Thomas, & Baeyens, 2001) and incentive or instrumental learning (Berridge, 2001; Dickinson & Balleine, 1994, 1995; Thorndike, 1898), the extent to which an outcome is desirable is determined by the affective experiences associated with the obtainment of the outcome. Hereby, relatively pleasurable experiences associated with specific outcomes allow these outcomes to serve as incentives for subsequent actions that are perceived as instrumental in obtaining these outcomes (Dickinson & Balleine, 1995). Recent research on the consolidation of ideomotor and incentive learning has indicated that affect can function as a feature of an action-outcome relationship. First, repeated experiences with relationships between actions and affective (positive vs. negative) action outcomes cause individuals to automatically select actions that produce positive and negative action outcomes (Beckers, de Houwer, & Eelen, 2002; Lavender & Hommel, 2007; Eder, Müsseler, & Hommel, 2012). Furthermore, such action-outcome learning eventually can become functional in biasing the individual’s motivational action orientation, such that actions are selected in the service of approaching positive outcomes and avoiding negative outcomes (Eder & Hommel, 2013; Eder, Rothermund, De Houwer & Hommel, 2015; Marien, Aarts & Custers, 2015).

This line of research suggests that people are able to predict their actions’ affective outcomes and bias their action selection accordingly through repeated experiences with the action-outcome relationship. Extending this combination of ideomotor and incentive learning to the domain of individual differences in implicit motivational dispositions and action selection, it can be hypothesized that implicit motives could predict and modulate action selection when two criteria are met. First, implicit motives would need to predict affective responses to stimuli that serve as outcomes of actions. Second, the action-outcome relationship between a specific action and this motive-congruent (dis)incentive would need to be learned through repeated experience.

According to motivational field theory, facial expressions can induce motive-congruent affect and thereby serve as motive-related incentives (Schultheiss, 2007; Stanton, Hall, & Schultheiss, 2010). As people with a high implicit need for power (*n*Power) hold a desire to influence, control and impress others (Fodor, 2010), they respond relatively positively to faces signaling submissiveness. This notion is corroborated by research showing that *n*Power predicts greater activation of the reward circuitry after viewing faces signaling submissiveness (Schultheiss & Schiepe-Tiska, 2013), as well as increased attention towards faces signaling submissiveness (Schultheiss & Hale, 2007; Schultheiss, Wirth, Waugh, Stanton, Meier, & Reuter-Lorenz, 2008). Indeed, previous research has indicated that the relationship between *n*Power and motivated actions towards faces signaling submissiveness can be susceptible to learning effects (Schultheiss & Rohde, 2002; Schultheiss, Wirth, Torges, Pang, Villacorta, & Welsh, 2005a). For example, *n*Power predicted response speed and accuracy after actions had been learned to predict faces signaling submissiveness in an acquisition phase (Schultheiss, Pang, Torges, Wirth, & Treynor, 2005b). Empirical support, then, has been obtained for both the idea that (1) implicit motives relate to stimuli-induced affective responses and (2) that implicit motives’ predictive capabilities can be modulated by repeated experiences with the action-outcome relationship. Consequently, for people high in *n*Power, an action predicting submissive faces would be expected to become increasingly more positive and hence increasingly more likely to be selected as people learn the action-outcome relationship, while the opposite would be true for actions predicting dominant faces as action outcomes.

## The present research

To test the proposed role of implicit motives (here specifically the need for power) in predicting action selection after action-outcome learning, we developed a novel task in which an individual repeatedly (and freely) decides to press one of two buttons. Each button leads to a different outcome, namely the presentation of a submissive or dominant face, respectively. This procedure is repeated 80 times to allow participants to learn the action-outcome relationship. As the actions will not initially be represented in terms of their outcomes, due to a lack of established history, *n*Power is not expected to immediately predict action selection. However, as participants’ history with the action-outcome relationship increases over trials, we expect *n*Power to become a stronger predictor of action selection in favor of the predicted motive-congruent incentivizing outcome. We report two studies to examine these expectations.

Study 1 aimed to offer an initial test of our ideas. Specifically, employing a within-subject design, participants repeatedly decided to press one of two buttons that were followed by a submissive or dominant face, respectively. This procedure thus allowed us to examine the extent to which *n*Power predicts action selection in favor of the predicted motive-congruent incentive as a function of the participant’s history with the action-outcome relationship. In addition, for exploratory purpose, Study 1 included a power manipulation for half of the participants. The manipulation involved a recall procedure of past power experiences that has frequently been used to elicit implicit motive-congruent behavior (e.g., Slabbinck, de Houwer, & van Kenhove, 2013; Woike, Bender, & Besner, 2009). Accordingly, we could explore whether the hypothesized interaction between *n*Power and history with the action-outcome relationship predicting action selection in favor of the predicted motive-congruent incentivizing outcome is conditional on the presence of power recall experiences.

## Study 1

### Method

#### Participants and design

Study 1 employed a stopping rule of at least 40 participants per condition, with additional participants being included if they could be found within the allotted time period. This resulted in eighty-seven students (40 female) with an average age of 22.32 years (SD = 4.21) participating in the study in exchange for a monetary compensation or partial course credit. Participants were randomly assigned to either the power (*n* = 43) or control (*n* = 44) condition.

#### Materials and procedure

The study started with the Picture Story Exercise (PSE); the most commonly used task for measuring implicit motives (Schultheiss, Yankova, Dirlikov, & Schad, 2009). The PSE is a reliable, valid and stable measure of implicit motives which is susceptible to experimental manipulation and has been used to predict a multitude of different motive-congruent behaviors (Latham & Piccolo, 2012; Pang, 2010; Ramsay & Pang, 2013; Pennebaker & King, 1999; Schultheiss & Pang, 2007; Schultheiss & Schultheiss, 2014). Importantly, the PSE shows no correlation with explicit measures (Köllner & Schultheiss, 2014; Schultheiss & Brunstein, 2001; Spangler, 1992). During this task, participants were shown six pictures of ambiguous social scenarios depicting, respectively, a ship captain and passenger; two trapeze artists; two boxers; two women in a laboratory; a couple by a river; a couple in a nightclub. These pictures have frequently been used to assess implicit motives and are the most strongly recommended pictorial stimuli (Pang & Schultheiss, 2005; Schultheiss & Pang, 2007). Pictures were presented in a random order for 10 s each. After each picture, participants had 2–4 min to write an imaginative story related to the picture’s content.

In accordance with Winter’s (1994) *Manual for scoring motive imagery in running text*, power motive imagery (*n*Power) was scored whenever the participant’s stories mentioned any strong and/or forceful actions with an inherent impact on other people or the world at large; attempts to control or regulate others; attempts to influence, persuade, convince, make or prove a point; provision of unsolicited help, advice or support; attempts to impress others or the world at large; (concern about) fame, prestige or reputation; or any strong emotional reactions in one person or group of people to the intentional actions of another. The condition-blind rater had previously obtained a confidence agreement exceeding 0.85 with expert scoring (Winter, 1994). A second condition-blind rater with similar expertise independently scored a random quarter of the stories (inter-rater reliability: *r* = 0.95). The absolute number of power motive images as assessed by the first rater (*M* = 4.62; SD = 3.06) correlated significantly with story length in words (*M* = 543.56; SD = 166.24), *r*(85) = 0.61, *p* < 0.01. In accordance with recommendations (Schultheiss & Pang, 2007), a regression for word count was therefore conducted, whereby *n*Power scores were converted to standardized residuals.

After the PSE, participants in the power condition were given 2–4 min to write down a story about an event where they had dominated the situation and had exercised control over others. This recall procedure is often used to elicit implicit motive-congruent behavior (e.g., Slabbinck et al., 2013; Woike et al., 2009). The recall procedure was omitted in the control condition.

**Fig. 1:**
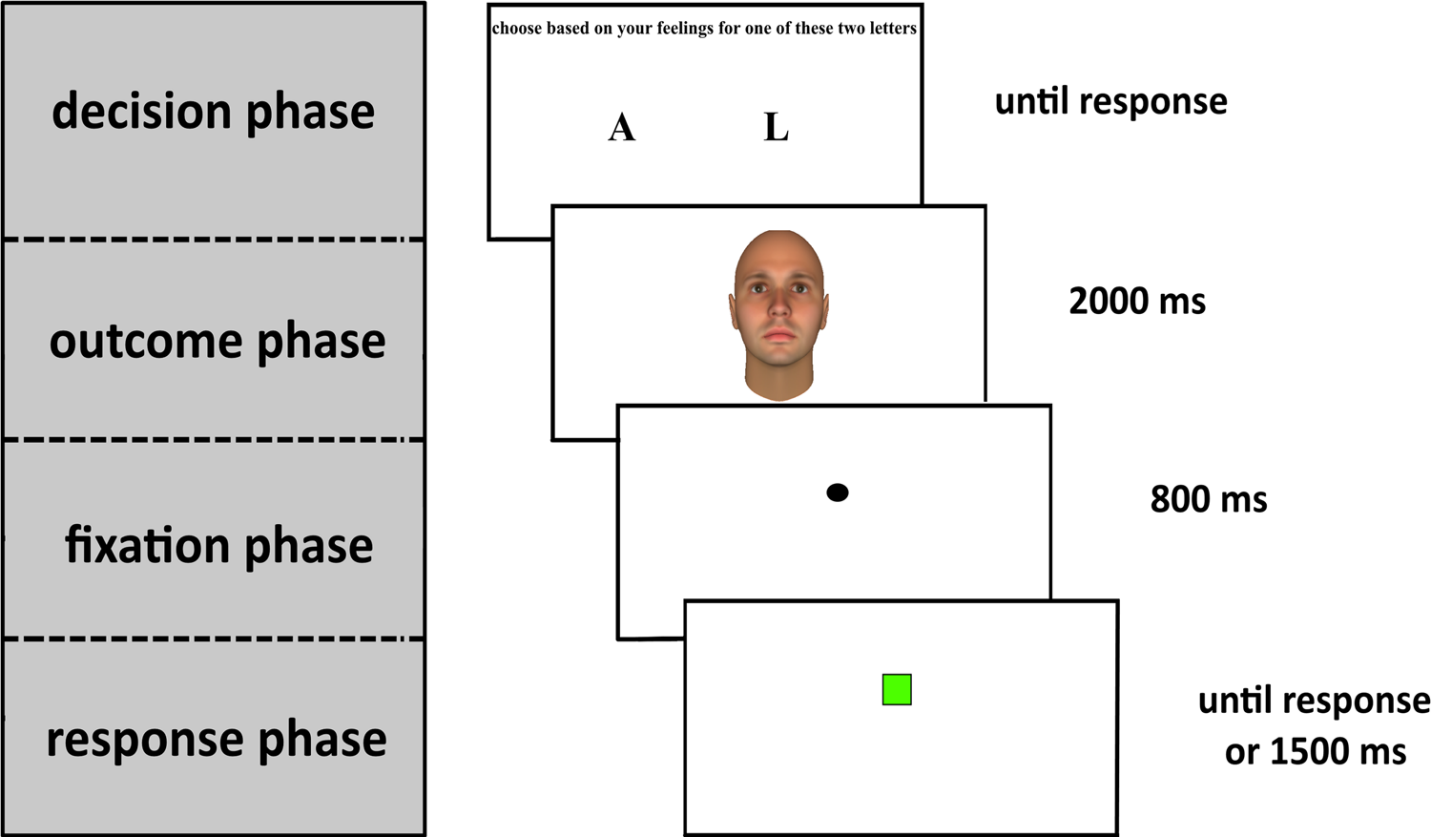
Procedure of one trial in the Decision-Outcome Task

Subsequently, participants partook in the newly developed Decision-Outcome Task (see Fig. 1). This task consisted of six practice and 80 critical trials. Each trial allowed participants an unlimited amount of time to freely decide between two actions, namely to press either a left or right key (i.e., the A or L button on the keyboard). Each key press was followed by the presentation of a picture of a Caucasian male face with a direct gaze, of which participants were instructed to meet the gaze. Faces were taken from the Dominance Face Data Set (Oosterhof & Todorov, 2008), which consists of computer-generated faces manipulated in perceived dominance with FaceGen 3.1 software. Two versions (one version two standard deviations *below* and one version two standard deviations *above* the mean dominance level) of six different faces were selected. These versions constituted the submissive and dominant faces, respectively. The decision to press left or right always led to either a randomly without replacement selected submissive or a randomly without replacement selected dominant face respectively. Which key press led to which face type was counter-balanced between participants.

Faces were shown for 2000 ms, after which an 800 ms black and circular fixation point was shown at the same screen location as had previously been occupied by the region between the faces’ eyes. This was followed by a randomly colored square or circle, shown for 1500 ms at the same location. Color randomization covered the whole color spectrum, except for values too difficult to distinguish from the white background (i.e., too close to white). Squares and circles were presented equally in a randomized order, with participants having to press the G button on the keyboard for squares and refrain from responding for circles. This fixation element of the task served to incentivize properly meeting the faces’ gaze, as the response-relevant stimuli were presented on spatially congruent locations. In the practice trials, participants’ responses or lack thereof were followed by accuracy feedback. After the square or circle (and subsequent accuracy feedback) had disappeared, a 500-millisecond pause was employed, followed by the next trial starting anew. Having completed the Decision-Outcome Task, participants were presented with several 7-point Likert scale control questions and demographic questions (see Tables 1 and 2 respectively in the supplementary online material).

#### Preparatory data analysis

Based on a priori established exclusion criteria, eight participants’ data were excluded from the analysis. For two participants, this was due to a combined score of three or lower on the control questions “How motivated were you to perform as well as possible during the decision task?” and “How important did you think it was to perform as well as possible during the decision task?”, on Likert scales ranging from 1 (*not motivated/important at all*) to 7 (*very motivated/important*). The data of four participants were excluded because they pressed the same button on more than 95 % of the trials, and two other participants’ data were excluded because they pressed the same button on 90 % of the first 40 trials. Other a priori exclusion criteria did not result in data exclusion.

## Results

### Power motive

**Fig. 2:**
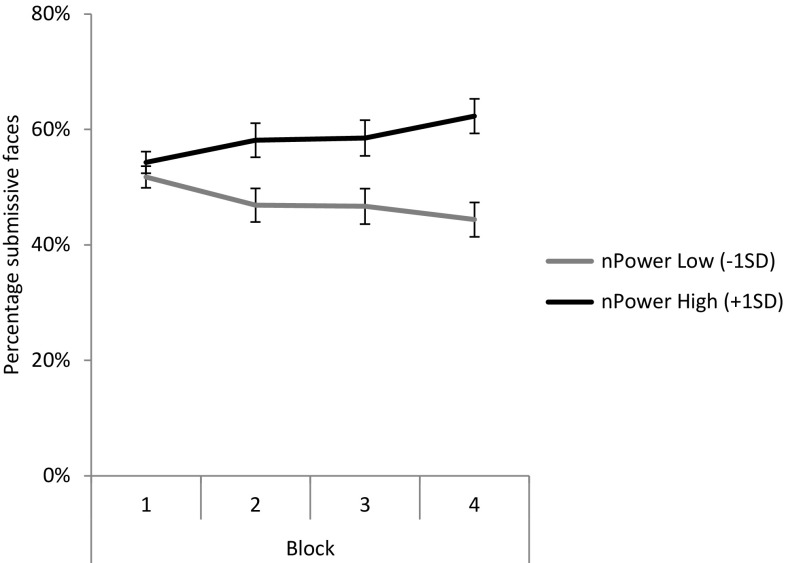
Estimated marginal means of choices leading to submissive (vs. dominant) faces as a function of block and *n*Power collapsed across recall manipulations. *Error bars* represent standard errors of the mean

We hypothesized that the implicit need for power (*n*Power) would predict the decision to press the button leading to the motive-congruent incentive of a submissive face after this action-outcome relationship had been experienced repeatedly. In accordance with commonly used practices in repetitive decision-making designs (e.g., Bowman, Evans, & Turnbull, 2005; de Vries, Holland, & Witteman, 2008), decisions were examined in four blocks of 20 trials. These four blocks served as a within-subjects variable in a general linear model with recall manipulation (i.e., power versus control condition) as a between-subjects factor and *n*Power as a between-subjects continuous predictor. We report the multivariate results as the assumption of sphericity was violated, *χ* = 15.49, *ε* = 0.88, *p* = 0.01. First, there was a main effect of *n*Power,^1^ *F*(1, 76) = 12.01, *p* < 0.01, $\upeta_{\text{p}}^{\text{2}}$ = 0.14. Furthermore, in line with expectations, the analysis yielded a significant interaction effect of *n*Power with the four blocks of trials,^2^ *F*(3, 73) = 7.00, *p* < 0.01, $\upeta_{\text{p}}^{\text{2}}$ = 0.22. Finally, the analyses yielded a three-way interaction between blocks, *n*Power and recall manipulation that did not reach the conventional level of significance,^3^ *F*(3, 73) = 2.66, *p* = 0.055, $\upeta_{\text{p}}^{\text{2}}$ = 0.10. Figure 2 presents the percentage of action choices leading to submissive (vs. dominant) faces as a function of block and *n*Power collapsed across recall manipulations (see Figures S1 and S2 in supplementary online material for figures per recall manipulation).

Conducting the aforementioned analysis separately for the two recall manipulations revealed that the interaction effect between *n*Power and blocks was significant in both the power, *F*(3, 34) = 4.47, *p* = 0.01, $\upeta_{\text{p}}^{\text{2}}$ = 0.28, and control condition, *F*(3, 37) = 4.79, *p* = 0.01, $\upeta_{\text{p}}^{\text{2}}$ = 0.28. Interestingly, this interaction effect followed a linear trend for blocks in the power condition, *F*(1, 36) = 13.65, *p* < 0.01, $\upeta_{\text{p}}^{\text{2}}$ = 0.28, but not in the control condition, *F*(1, 39) = 2.13, *p* = 0.15, $\upeta_{\text{p}}^{\text{2}}$ = 0.05. The main effect of *n*Power was significant in both conditions, *p*s ≤ 0.02. Taken together, then, the data suggest that the power manipulation was not required for observing an effect of *n*Power, with the only between-manipulations difference constituting the effect’s linearity.

### Additional analyses

We conducted several additional analyses to assess the extent to which the aforementioned predictive relations could be considered implicit and motive-specific. Based on a 7-point Likert scale control question that asked participants about the extent to which they preferred the pictures following either the left versus right key press (recoded depending on counterbalance condition), a linear regression analysis indicated that *n*Power did not predict people’s reported preferences, *t* = 1.05, *p* = 0.297. Adding this measure of explicit picture preference to the aforementioned analyses did not change the significance of *n*Power’s main or interaction effect with blocks (*p*s < 0.01), nor did this factor interact with blocks and/or *n*Power, *F*s < 1, suggesting that *n*Power’s effects occurred irrespective of explicit preferences.^4^ Furthermore, replacing *n*Power as predictor with either *n*Achievement or *n*Affiliation revealed no significant interactions of said predictors with blocks, *F*s(3, 75) ≤ 1.92, *p*s ≥ 0.13, indicating that this predictive relation was specific to the incentivized motive.

A prior investigation into the predictive relation between *n*Power and learning effects (Schultheiss et al., 2005b) observed significant effects only when participants’ sex matched that of the facial stimuli. We therefore explored whether this sex-congruency effect was also present here. As we used only male faces, the sex-congruency effect would entail a three-way interaction between *n*Power, blocks and sex with the effect being strongest for males. This three-way interaction did not, however, reach significance, *F* < 1, indicating that the aforementioned effects, *p*s < 0.01, did not depend on sex-congruency. Still, some effects of sex were observed, but none of these related to the learning effect, as indicated by a lack of significant interactions including blocks and sex. Hence, these results are only discussed in the supplementary online material.

## Discussion

Despite many studies indicating that implicit motives can predict which actions people choose to perform, less is known about how this action selection process arises. We argue that establishing an action-outcome relationship between a specific action and an outcome with motive-congruent (dis)incentive value can allow implicit motives to predict action selection (Dickinson & Balleine, 1994; Eder & Hommel, 2013; Schultheiss et al., 2005b). The first study supported this idea, as the implicit need for power (*n*Power) was found to become a stronger predictor of action selection as the history with the action-outcome relationship increased. This effect was observed irrespective of whether participants’ *n*Power was first aroused by means of a recall procedure.

It is important to note that in Study 1, submissive faces were used as motive-congruent incentives, while dominant faces were used as motive-congruent disincentives. As both of these (dis)incentives could have biased action selection, either together or separately, it is as of yet unclear to which extent *n*Power predicts action selection based on experiences with actions resulting in incentivizing or disincentivizing outcomes. Ruling out this issue allows for a more precise understanding of how *n*Power predicts action selection towards and/or away from the predicted motive-related outcomes after a history of action-outcome learning. Accordingly, Study 2 was conducted to further investigate this question by manipulating between participants whether actions led to submissive versus dominant, neutral versus dominant, or neutral versus submissive faces. The submissive versus dominant condition is similar to Study 1′s control condition, thus offering a direct replication of Study 1. However, from the perspective of the need for power, the second and third conditions can be conceptualized as avoidance and approach conditions, respectively.

## Study 2

### Method

#### Participants and design

Following Study 1’s stopping rule, one hundred and twenty-one students (82 female) with an average age of 21.41 years (SD = 3.05) participated in the study in exchange for a monetary compensation or partial course credit. Participants were randomly assigned to either the approach (*n* = 41), avoidance (*n* = 41) or control (*n* = 40) condition.

#### Materials and procedure

Study 2 was used to investigate whether Study 1’s results could be attributed to an approach towards the submissive faces due to their incentive value and/or an avoidance of the dominant faces due to their disincentive value. This study therefore largely mimicked Study 1’s protocol,^5^ with only three divergences. First, the power manipulation was omitted from all conditions. This was done as Study 1 indicated that the manipulation was not required for observing an effect. Furthermore, this manipulation has been found to increase approach behavior and hence may have confounded our investigation into whether Study 1’s results constituted approach and/or avoidance behavior (Galinsky, Gruenfeld, & Magee, 2003; Smith & Bargh, 2008).

Second, the approach and avoidance conditions were added, which used different faces as outcomes during the Decision-Outcome Task. The faces used by the approach condition were either submissive (i.e., two standard deviations *below* the mean dominance level) or neutral (i.e., mean dominance level). Conversely, the avoidance condition used either dominant (i.e., two standard deviations *above* the mean dominance level) or neutral faces. The control condition used the same submissive and dominant faces as had been used in Study 1. Hence, in the approach condition, participants could decide to approach an incentive (viz., submissive face), whereas they could decide to avoid a disincentive (viz., dominant face) in the avoidance condition and do both in the control condition.

Third, after completing the Decision-Outcome Task, participants in all conditions proceeded to the BIS-BAS questionnaire, which measures explicit approach and avoidance tendencies and had been added for explorative purposes (Carver & White, 1994). It is possible that dominant faces’ disincentive value only leads to avoidance behavior (i.e., more actions towards other faces) for people relatively high in explicit avoidance tendencies, while the submissive faces’ incentive value only leads to approach behavior (i.e., more actions towards submissive faces) for people relatively high in explicit approach tendencies. This exploratory questionnaire served to investigate this possibility. The questionnaire consisted of 20 statements, which participants responded to on a 4-point Likert scale ranging from 1 (*not true for me at all*) to 4 (*completely true for me*). The Behavioral Inhibition Scale (BIS) comprised seven questions (e.g., “I worry about making mistakes”; *α* = 0.75). The Behavioral Activation Scale (BAS) comprised thirteen questions (*α* = 0.79) and consisted of three subscales, namely the Reward Responsiveness (BASR; *α* = 0.66; e.g., “It would excite me to win a contest”), Drive (BASD; *α* = 0.77; e.g., “I go out of my way to get things I want”) and Fun Seeking subscales (BASF; *α* = 0.64; e.g., “I crave excitement and new sensations”).

#### Preparatory data analysis

Based on a priori established exclusion criteria, five participants’ data were excluded from the analysis. Four participants’ data were excluded because they pressed the same key on more than 95 % of the trials. One other participant’s data were excluded due to a consistent response pattern (i.e., minimal descriptive complexity of “40 times AL”).

## Results

### Power motive

**Fig. 3:**
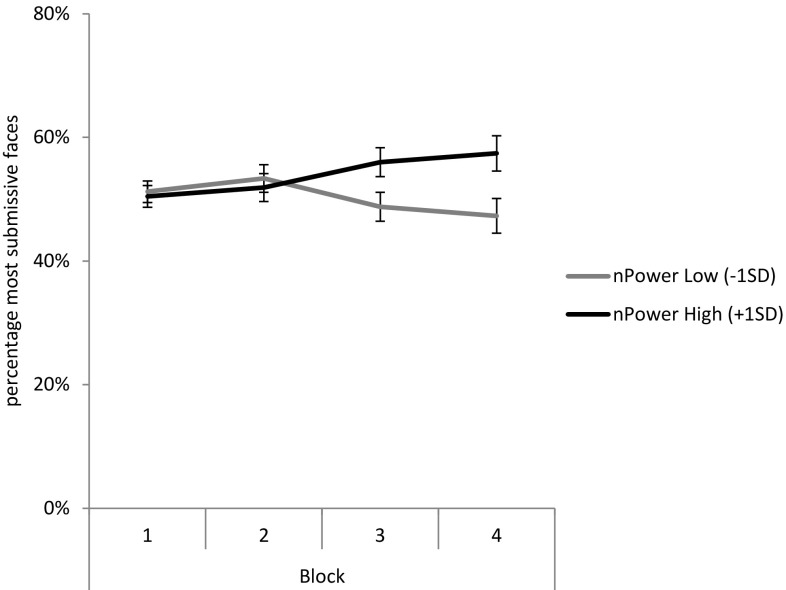
Estimated marginal means of choices leading to most submissive (vs. most dominant) faces as a function of block and *n*Power collapsed across the conditions in Study 2. *Error bars* represent standard errors of the mean

Study 2 sought to investigate whether *n*Power could predict the selection of actions based on outcomes that were either motive-congruent incentives (approach condition) or disincentives (avoidance condition) or both (control condition). To compare the different stimuli manipulations, we coded responses in accordance with whether they related to the most dominant (i.e., dominant faces in avoidance and control condition, neutral faces in approach condition) or most submissive (i.e., submissive faces in approach and control condition, neutral faces in avoidance condition) available option. We report the multivariate results because the assumption of sphericity was violated, *χ* = 23.59, *ε* = 0.87, *p* < 0.01. The analysis showed that *n*Power significantly interacted with blocks to predict decisions leading to the most submissive (or least dominant) faces,^6^ *F*(3, 108) = 4.01, *p* = 0.01, $\upeta_{\text{p}}^{\text{2}}$ = 0.10. Furthermore, no three-way interaction was observed including the stimuli manipulation (i.e., avoidance vs. approach vs. control condition) as factor, *F*(6, 216) = 0.19, *p* = 0.98, $\upeta_{\text{p}}^{\text{2}}$ = 0.01. Lastly, the two-way interaction between *n*Power and stimuli manipulation approached significance, *F*(1, 110) = 2.97, *p* = 0.055, $\upeta_{\text{p}}^{\text{2}}$ = 0.05. As this between-conditions difference was, however, neither significant, related to nor challenging the hypotheses, it is not discussed further. Figure 3 displays the mean percentage of action choices leading to the most submissive (vs. most dominant) faces as a function of block and *n*Power collapsed across the stimuli manipulations (see Figures S3, S4 and S5 in the supplementary online material for a display of these results per condition).

### Behavioral inhibition and activation scales

Before conducting the explorative analyses on whether explicit inhibition or activation tendencies affect the predictive relation between *n*Power and action selection, we examined whether participants’ responses on any of the behavioral inhibition or activation scales were affected by the stimuli manipulation. Separate ANOVA’s indicated that this was not the case, *F*s ≤ 1.23, *p*s ≥ 0.30. Next, we added the BIS, BAS or any of its subscales separately to the aforementioned repeated-measures analyses. These analyses did not reveal any significant predictive relations involving *n*Power and said (sub)scales, *p*s ≥ 0.10, except for a significant four-way interaction between blocks, stimuli manipulation, *n*Power and the Drive subscale (BASD), *F*(6, 204) = 2.18, *p* = 0.046, $\upeta_{\text{p}}^{\text{2}}$ = 0.06. Splitting the analyses by stimuli manipulation did not yield any significant interactions involving both *n*Power and BASD, *p*s ≥ 0.17. Hence, although the conditions observed differing three-way interactions between *n*Power, blocks and BASD, this effect did not reach significance for any specific condition. The interaction between participants’ *n*Power and established history regarding the action-outcome relationship therefore appears to predict the selection of actions both towards incentives and away from disincentives irrespective of participants’ explicit approach or avoidance tendencies.

### Additional analyses

In accordance with the analyses for Study 1, we again employed a linear regression analysis to investigate whether *n*Power predicted people’s reported preferences for pictures following the pressing of either button, which was not the case, *t* < 1. Adding this measure of explicit picture preferences to the aforementioned analyses again did not change the significance of *n*Power’s interaction effect with blocks, *p* = 0.01, nor did this factor interact with blocks or *n*Power, *F*s < 1, suggesting that *n*Power’s effects occurred irrespective of explicit preferences. Furthermore, replacing *n*Power as predictor with either *n*Achievement or *n*Affiliation again revealed no significant interactions of said predictors with blocks, *F*s(3,112) ≤ 1.42, *p*s ≥ 0.12, indicating that this predictive relation was specific to the incentivized motive. Lastly, we again observed no significant three-way interaction including *n*Power, blocks and participants’ sex, *F* < 1, nor were the effects including sex as denoted in the supplementary material for Study 1 replicated, *F*s < 1.

## General discussion

Building on a wealth of research showing that implicit motives can predict many different types of behavior, the present study set out to examine the potential mechanism by which these motives predict which specific behaviors people decide to engage in. We argued, based on theorizing regarding ideomotor and incentive learning (Dickinson & Balleine, 1995; Eder et al., 2015; Hommel et al., 2001), that previous experiences with actions predicting motive-congruent incentives are likely to render these actions more positive themselves and hence make them more likely to be selected. Accordingly, we investigated whether the implicit need for power (*n*Power) would become a stronger predictor of deciding to execute one over another action (here, pressing different buttons) as people established a greater history with these actions and their subsequent motive-related (dis)incentivizing outcomes (i.e., submissive versus dominant faces). Both Studies 1 and 2 supported this idea. Study 1 demonstrated that this effect occurs without the need to arouse *n*Power in advance, while Study 2 showed that the interaction effect of *n*Power and established history on action selection was due to both the submissive faces’ incentive value and the dominant faces’ disincentive value. Taken together, then, *n*Power seems to predict action selection as a result of incentive processing of faces that are represented as action-outcomes.

The present demonstration that implicit motives predict actions after they have become associated, by means of action-outcome learning, with faces differing in dominance level concurs with evidence collected to test central aspects of motivational field theory (Stanton et al., 2010). This theory argues, amongst others, that *n*Power predicts the incentive value of faces diverging in signaled dominance level. Studies that have supported this notion have shown that *n*Power is positively associated with the recruitment of the brain’s reward circuitry (especially the dorsoanterior striatum) after viewing relatively submissive faces (Schultheiss & Schiepe-Tiska, 2013), and predicts implicit learning as a result of, recognition speed of, and attention towards faces diverging in signaled dominance level (Donhauser et al., 2015; Schultheiss & Hale, 2007; Schultheiss et al., 2005b, 2008). The current studies extend the behavioral evidence for this idea by observing similar learning effects for the predictive relationship between *n*Power and action selection.

Furthermore, it is important to note that the present studies followed the ideomotor principle to investigate the potential building blocks of implicit motives’ predictive effects on behavior. The ideomotor principle, according to which actions are represented in terms of their perceptual results, provides a sound account for understanding how action-outcome knowledge is acquired and involved in action selection (Hommel, 2013; Shin et al., 2010). Interestingly, recent research provided evidence that affective outcome information can be associated with actions and that such learning can direct approach versus avoidance responses to affective stimuli that were previously learned to follow from these actions (Eder et al., 2015). Thus far, research on ideomotor learning has mainly focused on demonstrating that action-outcome learning pertains to the binding of actions and neutral or affect laden events, while the question of how social motivational dispositions, such as implicit motives, interact with the learning of the affective properties of action-outcome relationships has not been addressed empirically. The present research specifically indicated that ideomotor learning and action selection might be influenced by *n*Power, thereby extending research on ideomotor learning to the realm of social motivation and behavior. Accordingly, the present findings offer a model for understanding and examining how human decision-making is modulated by implicit motives in general.

To further advance this ideomotor explanation regarding implicit motives’ predictive capabilities, future research could examine whether implicit motives can predict the occurrence of a bidirectional activation of action-outcome representations (Hommel et al., 2001). Specifically, it is as of yet unclear whether the extent to which the perception of the motive-congruent outcome facilitates the preparation of the associated action is susceptible to implicit motivational processes. Future research examining this possibility could potentially provide further support for the current claim of ideomotor learning underlying the interactive relationship between *n*Power and a history with the action-outcome relationship in predicting behavioral tendencies.

Beyond ideomotor theory, it is worth noting that although we observed an increased predictive relationship between *n*Power and action selection as the learning history increased, this does not necessarily mean that the establishment of a learning history is required for *n*Power to predict action selection. Outcome predictions can be enabled through methods other than action-outcome learning (e.g., telling people what will happen) and such manipulations may, consequently, yield similar effects. The hereby proposed mechanism may therefore not be the only such mechanism allowing for *n*Power to predict action selection.

It is also worth noting that the currently observed predictive relation between *n*Power and action selection is inherently correlational. Although this makes conclusions regarding causality problematic, it does indicate that the Decision-Outcome Task (DOT) could be perceived as an alternative measure of *n*Power. These studies, then, could be interpreted as evidence for convergent validity between the two measures. Somewhat problematically, however, the power manipulation in Study 1 did not yield an increase in action selection favoring submissive faces (as a function of established history). Hence, these results could be interpreted as a failure to establish causal validity (Borsboom, Mellenberg, & van Heerden, 2004). A potential reason for this may be that the current manipulation was too weak to significantly affect action selection. In their validation of the PA-IAT as a measure of *n*Power, for example, Slabbinck, de Houwer and van Kenhove (2011) set the minimum arousal manipulation duration at 5 min, whereas Woike et al., (2009) used a 10 min long manipulation. Considering that the maximal length of our manipulation was 4 min, participants may have been given insufficient time for the manipulation to take effect. Subsequent studies could examine whether increased action selection towards submissive faces is observed when the manipulation is employed for a longer period of time. Further studies into the validity of the DOT task (e.g., predictive and causal validity), then, could help the understanding of not just the mechanisms underlying implicit motives, but also the assessment thereof.

With such further investigations into this topic, a greater understanding may be gained regarding the ways in which behavior could be motivated implicitly to result in more positive outcomes. That is, important activities for which people lack sufficient motivation (e.g., dieting) may be more likely to be selected and pursued if these activities (or, at least, components of these activities) are made predictive of motive-congruent incentives. Finally, as congruence between motives and behavior has been associated with greater well-being (Pueschel, Schulte, & Michalak, 2011; Schüler, Job, Fröhlich, & Brandstätter, 2008), we hope that our studies will ultimately help provide a better understanding of how people’s health and happiness might be more effectively promoted by motivating individuals to selecting the actions that increase their well-being.

## Electronic supplementary material

Below is the link to the electronic supplementary material.

Supplementary material 1 (DOCX 40 kb)

## Footnotes

1 Conducting the same analyses without any data removal did not change the significance of these results. There was a significant main effect of *n*Power, *F*(1, 81) = 11.75, *p* < 0.01, $\upeta_{\text{p}}^{\text{2}}$ = 0.13, a significant interaction between *n*Power and blocks, *F*(3, 79) = 4.79, *p* < 0.01, $\upeta_{\text{p}}^{\text{2}}$ = 0.15, and no significant three-way interaction between *n*Power, blocks andrecall manipulation, *F*(3, 79) = 1.44, *p* = 0.24, $\upeta_{\text{p}}^{\text{2}}$ = 0.05.

2 As an alternative analysis, we calculated changes in action selection by multiplying the percentage of actions selected towards submissive faces per block with their respective linear contrast weights (i.e., −3, −1, 1, 3). This measurement correlated significantly with *n*Power, *R* = 0.38, 95 % CI [0.17, 0.55]. Correlations between *n*Power and actions selected per block were *R* = 0.10 [−0.12, 0.32], *R* = 0.32 [0.11, 0.50], *R* = 0.29 [0.08, 0.48], and *R* = 0.41 [0.20, 0.57], respectively.

3 This effect was significant if, instead of a multivariate approach, we had elected to apply a Huynh–Feldt correction to the univariate approach, *F*(2.64, 225) = 3.57, *p* = 0.02, $\upeta_{\text{p}}^{\text{2}}$ = 0.05.

4 A more detailed measure of explicit preferences had been conducted in a pilot study (*n* = 30). Participants were asked to rate each of the faces employed in the Decision-Outcome Task on how positively they experienced and attractive they considered each face on separate 7-point Likert scales. The interaction between face type (dominant vs. submissive) and *n*Power did not significantly predict evaluations, *F* < 1. *n*Power did show a significant main effect, *F*(1,27) = 6.74, *p* = 0.02, $\upeta_{\text{p}}^{\text{2}}$ = 0.20, indicating that people high in *n*Power generally rated other people’s faces more negatively. These data further support the idea that *n*Power does not relate to explicit preferences for submissive over dominant faces.

5 The number of power motive images (*M* = 4.04; SD = 2.62) again correlated significantly with story length in words (*M* = 561.49; SD = 172.49), *r*(121) = 0.56, *p* < 0.01, We therefore again converted the *n*Power score to standardized residuals after a regression for word count.

6 Conducting the same analyses without any data removal did not change the significance of the hypothesized results. There was a significant interaction between *n*Power and blocks, *F*(3, 113) = 4.14, *p* = 0.01, $\upeta_{\text{p}}^{\text{2}}$ = 0.10, and no significant three-way interaction between *n*Power, blocks and stimuli manipulation, *F*(6, 226) = 0.23, *p* = 0.97, $\upeta_{\text{p}}^{\text{2}}$ = 0.01. Conducting the alternative analysis, whereby changes in action selection were calculated by multiplying the percentage of actions selected towards submissive faces per block with their respective linear contrast weights (i.e., −3, −1, 1, 3), again revealed a significant correlation between this measurement and *n*Power, *R* = 0.30, 95 % CI [0.13, 0.46]. Correlations between *n*Power and actions selected per block were *R* = −0.01 [−0.20, 0.17], *R* = −0.04 [−0.22, 0.15], *R* = 0.21 [0.03, 0.38], and *R* = 0.25 [0.07, 0.41], respectively.
