# Supplementary material for: The implicit power motive predicts action selection
Source: Psychol Res. 2016 Mar 23;81(3):560–70. doi: 10.1007/s00426-016-0768-z (PMC5397432; doi:10.1007/s00426-016-0768-z)
Supplement: Supplementary file 1 — Supplementary material 1 (DOCX 40 kb) [file 426_2016_768_MOESM1_ESM.docx]

**Supplementary Online Material**

**Control and demographic questions**

Table 1

*Questions and Anchors of the Control Questions with 7-point Likert Scales*

| Question | Anchors |
| --- | --- |
| How motivated were you to perform as well as possible during the decision task? | *not motivated at all* - *very motivated* |
| How important did you think it was to perform as well as possible during the decision task? | *not important at all* - *very important* |
| How difficult did you think it was to perform as well as possible during the decision task? | *not difficult at all* - *very difficult* |
| How did you decide which button to press? | *mostly by thinking rationally* - *mostly based on feelings* |
| Did you prefer pressing the A or L button? | *much preferred A – no preference – much preferred L* |
| Did you more frequently press the A or L button? | *A a lot more frequently – no difference – L a lot more frequently* |
| Did you, during the first element of the decision task, prefer watching the pictures that appeared after the A or after the L button? | *much preferred those after A– no preference – much preferred those after L* |
| Did you, during the decision task, press the G button with your left or right hand? | *(almost) always left hand – both equally – (almost) always right hand* |

*Note.* If three anchors are mentioned, the second anchor related to the neutral option on the 7-point Likert scale (“4”).

Table 2

*Questions and Answering Options of the Demographic and Other Questions*

| Question | Answering options | |
| --- | --- | --- |
| Did you previously partake in an experiment using the decision task with faces? | | *1: yes*  *2: no*  *3: not sure* |
| What is your age (in years)? | | *max. three characters* |
| Are you male or female? | | *1: male*  *2: female* |
| Are you a student at a university? | | *1: yes*  *2: no, at an applied university*  *3: no, not a student (anymore)* |
| What kind of compensation will you receive for taking part in this experiment? | | *1: money*  *2: partial course credit* |
| Is Dutch your native language? | | *1: yes*  *2: no* |
| Are you left- or right-handed? | | *1: left*  *2: right*  *3: both* |
| What do you think or suspect this research attempted to measure? | | *max. 500 characters* |
| Do you have any further remarks about the experiment or suggestions for improvement? | | *max. 500 characters* |

*Note.* The numbers before the answering options represent the order of appearance and were not shown in the study.

**Additional Figures Study 1**

*Figure S1*. Estimated marginal means of choices leading to submissive (vs. dominant) faces as a function of block and *n*Power in the power condition. Error bars represent Standard Errors of the mean.

*Figure S2*. Estimated marginal means of choices leading to submissive (vs. dominant) faces as a function of block and *n*Power in the control condition. Error bars represent Standard Errors of the mean.

**Additional Analyses Study 1**

An analysis including participants’ sex as an independent variable indicated a significant main effect of sex, *F*(1, 71) = 7.69, *p* = .01, $\eta_{p}^{2}$ = .10, with women (*M* = 57.21%, *SE* = 2.04) being more likely to select actions predictive of submissive faces than men (*M* = 49.28%, *SE* = 2.05). This main effect was qualified by a significant two-way interaction between sex and *n*Power, *F*(1, 71) = 4.21, *p* = .04, $\eta_{p}^{2}$ = .06, and a significant three-way interaction between said factors and recall manipulation, *F*(1, 71) = 5.13, *p* = .03, $\eta_{p}^{2}$ = .07. Splitting the analyses by recall manipulation revealed a main effect of sex only in the power condition, *F*(1, 34) = 8.01, *p* = .01, $\eta_{p}^{2}$ = .19 (*F* < 1 in control condition), and a significant interaction between sex and *n*Power only in the control condition, *F*(1, 37) = 13.62, *p* < .01, $\eta_{p}^{2}$ = .27 (*F* < 1 in power condition). Splitting the analyses instead by sex revealed a significant interaction between recall manipulation and *n*Power only for women, *F*(1, 33) = 5.47, *p* = .03, $\eta_{p}^{2}$ = .14, with this effect being non-significant for men, *F*(1,38) = 1.02, *p* = .32. Based on these analyses, it could be expected that women would show a stronger main effect of *n*Power specifically in the control condition. This effect was indeed observed, *F*(1, 15) = 22.25, *p* < .01, $\eta_{p}^{2}$ = .60 (*F* < 1 for men in same condition), with women low in *n*Power (i.e., *M* – 1*SD*) selecting the action towards a submissive faces less (*M* = 44.20%, *SE* = 3.60) than women high in *n*Power (i.e., *M* + 1*SD*; *M* = 70.21%, *SE* = 4.07). Note that the sample for which this effect was observed is relatively small, making conclusions regarding its generalizability relatively problematic.

**Additional Figures Study 2**

*Figure S3*. Estimated marginal means of choices leading to neutral (vs. dominant) faces as a function of block and *n*Power in the avoidance condition. Error bars represent Standard Errors of the mean.

*Figure S4*. Estimated marginal means of choices leading to submissive (vs. neutral) faces as a function of block and *n*Power in the approach condition. Error bars represent Standard Errors of the mean.

*Figure S5*. Estimated marginal means of choices leading to submissive (vs. dominant) faces as a function of Block and *n*Power in the control condition. Error bars represent Standard Errors of the mean.
